# Supplementary material for: Habitual Dietary Intake Affects the Altered Pattern of Gut Microbiome by Acarbose in Patients with Type 2 Diabetes
Source: Nutrients. 2021 Jun 19;13(6):2107. doi: 10.3390/nu13062107 (PMC8235473; doi:10.3390/nu13062107)
Supplement: Supplementary file 1 [file nutrients-13-02107-s001.zip › nutrients-1235279-supplementary.pdf]

Supplementary Figure S1

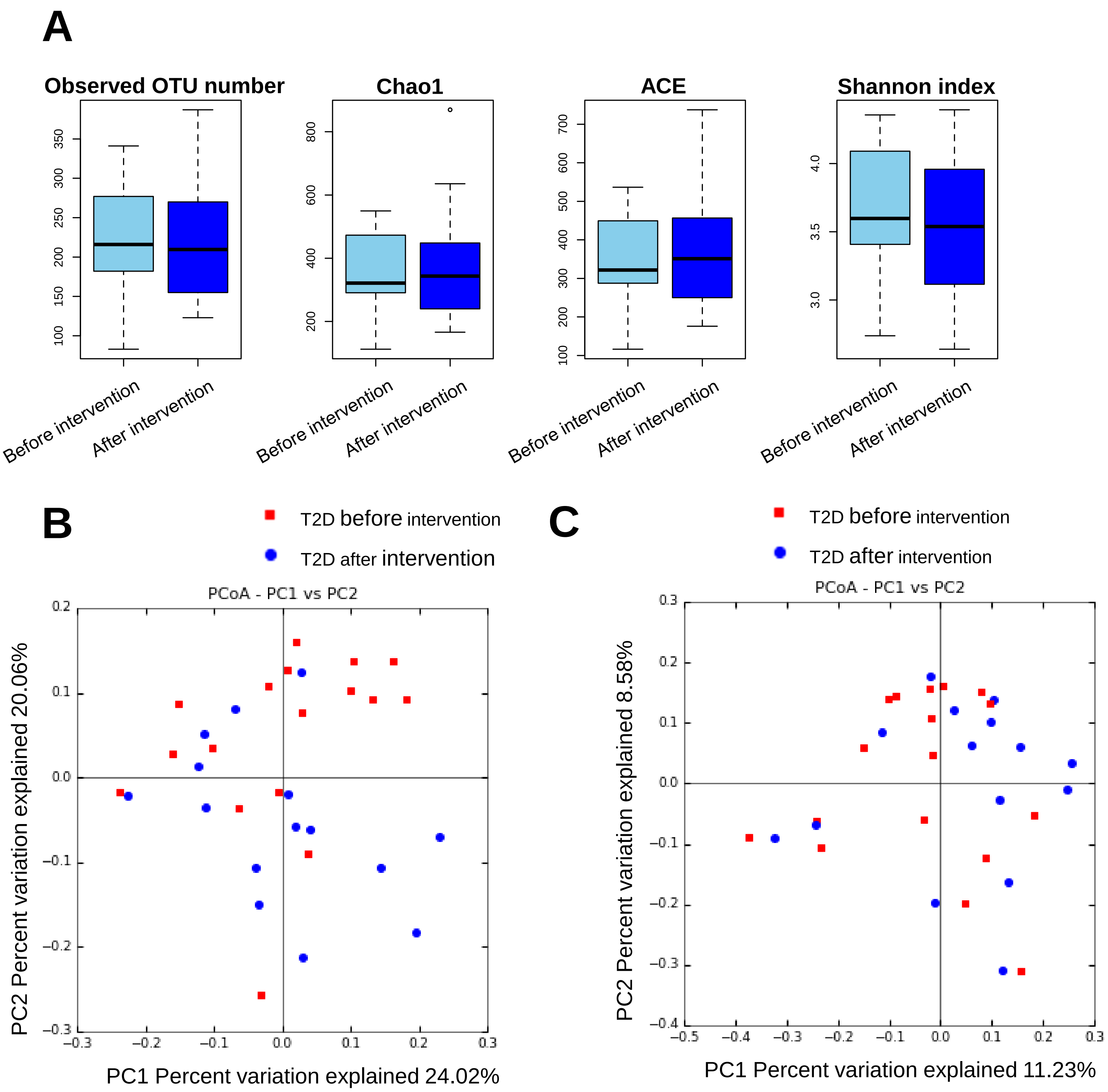

D

Category	No. subject	Weighted UniFrac		Unweighted UniFrac	
		R^2	*p*-value	R^2	*p*-value
T2D before intervention of acarbose vs T2D after intervention of acarbose	18	0.05	0.10	0.02	0.93

(A) Alpha diversity indexes. Comparisons of the observed and Chao 1-estimated operational taxonomic units (OTU number), the abundance-based coverage estimator (ACE), and the Shannon index of gut microbiome before and after the intervention of acarbose based on 16S analysis. Each box plot shows median, interquartile range, minimum, and maximum values. (B, C) Beta diversity indexes based on UniFrac analysis. Weighted (B) and unweighted (C) UniFrac-principal coordinate analysis (PCoA) of gut microbiome for patients with type 2 diabetes (T2D) before and after the intervention of acarbose based on 16S data. (D) Permutational multivariate analysis of variance of the gut microbiome samples between the two groups based on 16S data.

Supplementary Figure S2

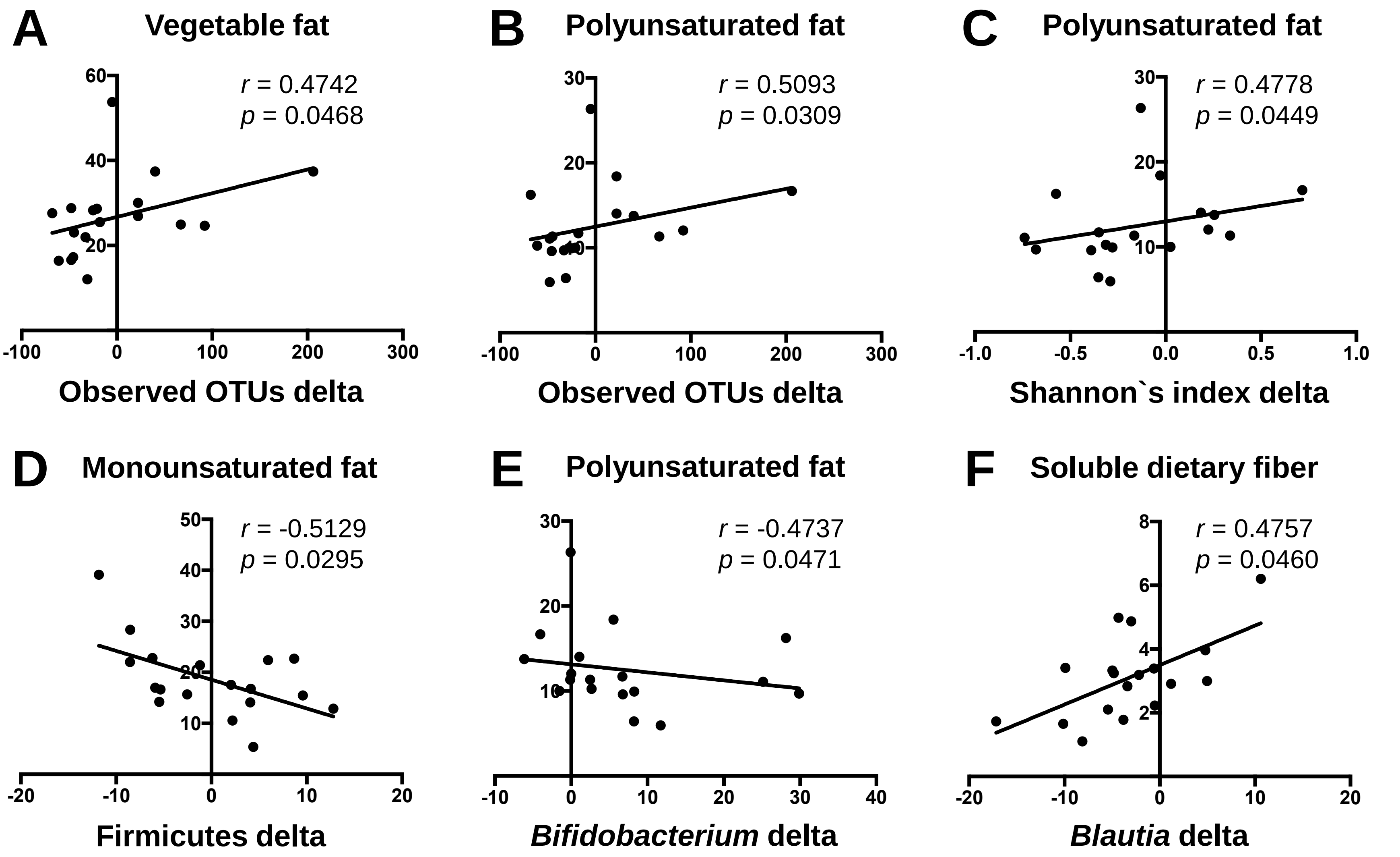

(A-F) Spearman's correlation between the relative abundance delta of microbial data and habitual dietary nutrient intake with statistical significance. X delta = X after intervention − X before intervention.

Supplementary Table S1

	Average relative abundance (%)	
**Phylum**	**Before intervention**	**After intervention**
Firmicutes	58.17 ± 2.38	58.08 ± 2.85
**Bacteroidetes ***	**24.98 ± 2.34**	**16.89 ± 1.65**
**Actinobacteria ***	**10.43 ± 2.36**	**18.17 ± 2.73**
Proteobacteria	2.53 ± 0.45	1.80 ± 0.29
Verrucomicrobia	1.60 ± 0.89	2.94 ± 1.19
Fusobacteria	0.51 ± 0.28	0.01 ± 0.003

Comparison of the relative abundance at the phylum level before and after the intervention of the acarbose based on 16S data. Phyla with an average relative abundance ≥0.1% in either of the two groups are represented. Data are represented as mean ± SEM. *p <0.05 based on the paired t test.

Supplementary Table S2

	Average relative abundance (%)	
**Genus**	**Before intervention**	**After intervention**
***Bifidobacterium* ***	**7.16 ± 2.26**	**14.09 ± 2.62**
***Bacteroides* ***	**13.61 ± 1.36**	**8.86 ± 1.07**
***Blautia* ***	**12.17 ± 1.75**	**9.02 ± 1.87**
***Prevotella* ***	**7.34 ± 2.53**	**4.24 ± 1.56**
***Eubacterium* ***	**2.48 ± 0.63**	**6.75 ± 1.85**
Faecalibacterium	5.45 ± 1.21	4.26 ± 0.76
Megamonas	4.82 ± 1.89	1.59 ± 0.78
Streptococcus	4.77 ± 1.50	4.75 ± 1.56
***Megasphaera* ***	**2.15 ± 0.59**	**3.91 ± 0.94**
Fusicatenibacter	1.94 ± 0.41	3.86 ± 1.69
Collinsella	2.90 ± 0.64	3.72 ± 0.79
***Clostridium* ***	**3.06 ± 0.46**	**1.77 ± 0.36**
Akkermansia	1.60 ± 0.89	2.94 ± 1.19
***Lactobacillus* ***	**0.89 ± 0.61**	**2.86 ± 0.84**
Subdoligranulum	1.98 ± 0.56	2.64 ± 0.65
Ruminococcus	2.40 ± 0.68	1.81 ± 0.50
Dorea	2.07 ± 0.42	1.74 ± 0.33
Parabacteroides	1.75 ± 0.37	1.51 ± 0.42
Alistipes	1.47 ± 0.51	1.61 ± 0.84
Acidaminococcus	1.13 ± 0.36	1.30 ± 0.46
Veillonella	0.80 ± 0.39	1.08 ± 0.52
***Phascolarctobacterium* ***	**0.93 ± 0.23**	**0.50 ± 0.15**
***Lachnoclostridium* ***	**0.92 ± 0.17**	**0.59 ± 0.12**
Anaerostipes	0.87 ± 0.39	0.68 ± 0.21
Escherichia	0.79 ± 0.30	0.67 ± 0.26
Holdemanella	0.73 ± 0.31	0.76 ± 0.37
Catenibacterium	0.68 ± 0.33	0.45 ± 0.23
Sutterella	0.65 ± 0.16	0.50 ± 0.14
Dialister	0.60 ± 0.27	0.47 ± 0.16
Roseburia	0.33 ± 0.10	0.57 ± 0.31
Oscillibacter	0.52 ± 0.14	0.50 ± 0.18
Fusobacterium	0.51 ± 0.28	0.01 ± 0.003

Comparison of the relative abundance at the genus level before and after the intervention of acarbose based on 16S data. Genera with an average relative abundance ≥0.5% in either of the two groups are represented. Data are represented as mean ± SEM. **p* <0.05 based on the paired t-test.

Supplementary Table S3

Dietary component	median	(range)
Carbohydrate, g	219.0	(115.5—442.7)
Sucrose, g	13.6	(1.0—36.2))
Soluble dietary fiber, g	3.1	(1.1—6.2)
Insoluble dietary fiber, g	8.6	(4.4—15.1)
Animal fat, g	21.7	(6.4—46.5)
Vegetable fat, g	26.2	(12.1—53.8)
Saturated fat, g	13.8	(4.1—23.6)
Monounsaturated fat, g	16.9	(5.4—39.1)
Polyunsaturated fat, g	11.3	(6.0—26.3)
Cholesterol,mg	387.8	(174.0—827.0)
Animal protein, g	44.8	(14.3—84.4)
Vegetable protein, g	28.5	(14.4—44.5)

Intake of each dietary component based on a Brief type self administered Diet History Questionnaire (BDHQ) among the total 18 subjects. Data are represented as median (range).

Supplementary Table S4

**Spearman`s correlation**		**Habitual dietary essential nutrient intake**											
**Item**	**Microbial data delta**	Carbohydrate	Sucrose	Soluble dietary fiber	Insoluble dietary fiber	Animal fat	Vegetable fat	Saturated fat	Monounsaturated fat	Polyunsaturated fat	Cholesterol	Animal protein	Vegetable protein
**Alpha diversity**	**Observed OTUs delta**	0.18	-0.35	0.17	0.19	-0.04	**0.47***	0.03	0.23	**0.51***	0.31	0.17	0.21
	**Shannon's index delta**	0.09	-0.23	0.11	0.08	0.07	0.42	0.19	0.33	**0.48***	0.32	0.18	0.08
**Phylum**	**Firmicutes delta**	-0.15	-0.08	-0.10	-0.11	-0.43	-0.24	-0.43	**-0.51***	-0.32	-0.38	-0.32	-0.06
	Bacteroidetes delta	-0.03	-0.19	-0.14	-0.12	0.14	0.28	0.23	0.33	0.28	0.16	-0.04	-0.16
	Actinobacteria delta	-0.04	0.20	0.12	0.11	-0.25	-0.31	-0.36	-0.38	-0.38	-0.26	-0.16	0.11
	Proteobacteria delta	-0.13	-0.15	-0.05	-0.15	0.26	-0.01	0.09	0.10	0.03	0.02	0.03	-0.18
**Genus**	***Bifidobacterium* delta**	-0.14	0.33	0.001	-0.03	-0.22	-0.44	-0.33	-0.41	**-0.47***	-0.29	-0.21	-0.03
	Bacteroides delta	0.11	0.04	0.22	0.15	-0.06	0.36	0.07	0.25	0.42	0.16	-0.01	0.10
	***Blautia* delta**	0.13	0.08	**0.48***	0.40	0.07	0.12	-0.15	0.16	0.39	0.21	0.20	0.33
	Prevotella delta	-0.14	-0.26	-0.22	-0.14	-0.19	0.02	-0.22	-0.16	-0.20	-0.32	-0.43	-0.20
	Eubacterium delta	0.11	0.20	-0.07	-0.04	-0.36	0.09	-0.01	-0.10	-0.31	-0.06	-0.21	-0.02

Z score		**Habitual dietary essential nutrient intake**											
**Item**	**Microbial data delta**	Carbohydrate	Sucrose	Soluble dietary fiber	Insoluble dietary fiber	Animal fat	Vegetable fat	Saturated fat	Monounsaturated fat	Polyunsaturated fat	Cholesterol	Animal protein	Vegetable protein
**Alpha diversity**	**Observed OTUs delta**	-1.48	1.20	0.25	0.51	1.28	0.73	1.28	1.38	1.21	0.97	0.65	1.49
	**Shannon's index delta**	-0.91	1.25	0.78	1.24	1.32	1.11	1.20	1.21	0.40	0.30	0.33	0.75
**Phylum**	**Firmicutes delta**	-0.20	-1.23	-1.63	-1.60	-1.47	-2.06	-0.98	-1.07	-0.48	-0.86	-0.78	-1.23
	Bacteroidetes delta	-0.72	0.16	1.12	1.42	0.68	1.11	0.66	0.73	-1.10	-0.92	-0.99	-0.24
	Actinobacteria delta	1.12	-0.43	-0.76	-1.28	-0.99	-1.56	-1.14	-1.31	0.59	0.48	0.38	-0.32
	Proteobacteria delta	-0.53	0.51	1.70	0.78	0.13	0.24	-0.02	-0.27	-1.23	-1.10	-0.51	-1.06
**Genus**	***Bifidobacterium* delta**	1.74	-0.68	-0.62	-1.14	-1.11	-1.68	-1.39	-1.76	-0.29	-0.37	-0.24	-1.14
	Bacteroides delta	0.37	0.31	0.15	0.69	0.68	0.81	1.04	1.00	0.52	0.72	0.91	0.91
	***Blautia* delta**	0.56	1.35	0.78	-0.32	0.88	0.47	0.95	0.18	1.96	2.24	2.28	1.08
	Prevotella delta	-1.06	-1.77	-0.47	-0.64	-1.23	-0.73	-0.65	-0.17	-1.35	-1.04	-1.41	-1.14
	Eubacterium delta	1.12	-0.68	-1.30	0.32	-0.19	-0.51	-0.95	0.07	-0.23	-0.43	-0.62	0.91

Spearman`s correlation of habitual dietary nutrient intake with the observed OTU number delta, Shannon index delta, and average relative abundance delta of the top four phyla and top five genera. *$p < 0.05$ based on the spearman’s rank correlation coefficient. Raw Z scores based on Spearman’s r in the analysis of correlation between habitual dietary nutrient intake and the observed OTU number delta, Shannon index delta, and average relative abundance delta of the top four phyla and top five genera. X delta = X after intervention − X before intervention.

Supplementary Table S5

Participants' clinical characteristics

No.	sex	age	dose of acarbose	duration	abdominal surgery	retinopathy	nephropathy	neuropathy	CVD	CAD	BMI (0w)	BMI (4w)	HbA1c (0w)	HbA1c (4w)
**A0**	M	70	150	13	No	NDR	1	No	No	No	22.7	22.7	7.2	7.1
**A1**	M	72	150	7	Yes	NDR	1	No	No	No	24.0	24.0	8	7.3
**A2**	F	71	150	9	No	NDR	1	No	No	No	23.5	23.5	7.6	7.5
**A3**	M	64	300	11	No	NDR	2	Yes	No	No	26.7	26.7	6.8	7.9
**A4**	F	73	300	13	No	SDR	1	No	No	No	24.2	24.2	6.6	7.1
**A5**	F	75	300	7	No	NDR	2	No	No	No	19.0	19.0	8	7.4
**A6**	F	73	150	5	No	NDR	1	No	No	No	22.2	22.2	8.7	7.6
**A8**	F	58	150	NA	No	NDR	1	No	No	No	26.8	26.8	6.9	6.7
**A9**	M	57	300	10	No	NDR	1	No	No	No	22.7	22.6	8.6	7.5
**A10**	F	57	300	6	No	NDR	1	No	No	No	23.9	23.4	6.8	6.5
**A11**	M	69	150	14	No	NDR	1	No	No	No	21.6	21.6	9.9	8.4
**A12**	M	64	150	14	No	NDR	1	Yes	No	No	23.0	23.0	6.9	6.8
**A13**	M	59	150	8	No	NDR	1	No	No	No	22.7	22.8	6.7	6.4
**A14**	M	48	150	28	No	NDR	1	No	No	No	35.9	35.9	7.9	7.6
**A15**	F	48	150	2	No	NDR	1	No	No	No	34.6	34.6	9.2	9.6
**A16**	F	70	150	13	No	NDR	1	No	No	No	21.9	21.5	7.1	6.9
**A17**	F	59	150	1	No	PDR	2	Yes	No	No	22.5	22.5	8	7.3
**A18**	M	74	150	19	No	PPDR	3	No	No	No	20.6	20.6	6.9	7.1

Participants' clinical characteristics

No.	SU	glinide	TZD	DPP4-i	metformin	SGLT2-i	insulin	ACE-i/ARB	statin	PPI	H2-blocker
**A0**	Yes	No	No	Yes	No	No	No	Yes	No	No	No
**A1**	Yes	No	No	Yes	Yes	No	No	No	No	No	No
**A2**	No	No	No	Yes	Yes	No	No	No	No	No	No
**A3**	Yes	No	No	Yes	Yes	Yes	Yes	Yes	No	No	No
**A4**	Yes	No	No	Yes	Yes	Yes	No	No	Yes	Yes	No
**A5**	No	No	No	Yes	Yes	Yes	No	No	Yes	No	No
**A6**	No	No	No	Yes	Yes	No	No	No	No	No	No
**A8**	No	No	No	Yes	Yes	No	No	No	Yes	No	No
**A9**	No	Yes	No	Yes	Yes	Yes	No	Yes	No	No	No
**A10**	Yes	No	No	Yes	Yes	No	No	No	No	No	No
**A11**	No	No	No	No	Yes	No	Yes	Yes	No	No	No
**A12**	No	No	No	Yes	Yes	No	No	Yes	No	No	No
**A13**	No	No	No	Yes	No	Yes	No	No	No	No	No
**A14**	Yes	No	Yes	Yes	Yes	Yes	No	Yes	No	No	No
**A15**	No	No	No	No	No	Yes	No	No	No	No	No
**A16**	No	No	No	No	Yes	Yes	No	No	No	No	No
**A17**	No	No	No	Yes	Yes	Yes	No	No	No	No	No
**A18**	No	No	No	Yes	No	Yes	No	Yes	No	Yes	No

Participants' dietary intake

No.	Carbohydrate	Sucrose	Soluble dietary fiber	Insoluble dietary fiber	Animal fat	Vegetable fat	Saturated fat	Monounsaturated fat	Polyunsaturated fat	Cholesterol	Animal protein	Vegetable protein
**A0**	173.4	2.4	2.1	5.6	6.4	12.1	4.1	5.4	6.4	173.7	14.3	24.4
**A1**	307.8	21.6	3.2	9.9	19.2	28.8	13.7	17.0	11.1	328.0	30.2	32.5
**A2**	235.1	20.4	5.0	13.1	21.7	28.7	15.5	17.6	10.0	336.2	51.7	29.5
**A3**	442.7	1.0	3.4	12.1	42.1	53.8	20.1	39.1	26.3	827.0	84.4	44.4
**A4**	229.7	36.2	3.3	9.7	18.7	28.3	14.4	15.5	9.9	383.3	39.8	32.3
**A5**	241.8	14.0	2.8	8.6	22.0	25.5	12.0	16.8	11.7	409.4	47.6	29.3
**A6**	208.2	29.0	3.4	9.4	21.7	23.1	11.8	15.7	11.3	308.4	32.4	28.1
**A8**	186.5	19.6	3.2	8.5	19.6	22.0	11.1	14.2	9.7	345.0	42.0	24.7
**A9**	299.0	14.9	1.7	5.8	18.8	37.4	14.7	21.4	13.7	392.3	22.9	27.5
**A10**	144.6	15.7	1.8	5.0	14.9	16.6	10.6	10.6	6.0	232.9	19.9	15.7
**A11**	239.2	4.4	6.2	15.1	35.2	30.1	13.9	22.4	18.4	651.1	75.8	39.8
**A12**	202.0	8.1	3.0	8.1	19.9	25.0	10.8	16.7	11.3	429.9	35.3	26.3
**A13**	320.4	32.3	4.9	11.7	40.7	27.7	19.2	22.8	16.2	674.9	75.8	37.3
**A14**	332.3	13.1	2.2	7.6	36.0	26.9	18.2	22.7	14.0	557.4	47.7	29.0
**A15**	115.5	3.8	1.7	4.5	22.5	17.3	12.0	12.9	9.6	282.9	37.6	17.8
**A16**	203.8	11.0	4.0	11.3	18.7	24.7	10.7	14.1	12.0	329.7	48.3	31.1
**A17**	147.4	8.2	1.1	4.4	46.5	16.4	23.6	22.0	10.3	472.9	56.0	14.4
**A18**	177.1	13.3	2.9	7.9	38.2	37.4	20.5	28.4	16.7	520.6	52.7	27.8

Clinical characteristics and dietary intake of the participants. Subject A7 was excluded from the present study due to the lack of dietary intake data. Intake of each dietary component is based on a Brief type self administered Diet History Questionnaire (BDHQ) among the total 18 subjects. Abbreviations: CVD = cerebrovascular disease; CAD = coronary artery disease; BMI = body mass index; HbA1c = hemoglobin A1c; SU = sulfonylurea; TZD = thiazolidine; DPP4-i = dipeptidyl peptidase 4 inhibitors; SGLT2-i = sodium glucose transporter 2 inhibitors; ACE-i = angiotensin converting enzyme inhibitors; ARB = angiotensin II receptor blockers; PPI = proton pump inhibitors
